# *Bacteroides thetaiotaomicron* Starch Utilization Promotes Quercetin Degradation and Butyrate Production by *Eubacterium ramulus*

**DOI:** 10.3389/fmicb.2019.01145

**Published:** 2019-05-29

**Authors:** Gina Paola Rodriguez-Castaño, Matthew R. Dorris, Xingbo Liu, Bradley W. Bolling, Alejandro Acosta-Gonzalez, Federico E. Rey

**Affiliations:** ^1^Grupo de Investigacion en Bioprospeccion (GIBP), Facultad de Ingenieria, Universidad de La Sabana, Chía, Colombia; ^2^Department of Food Science, University of Wisconsin-Madison, Madison, WI, United States; ^3^Department of Bacteriology, University of Wisconsin-Madison, Madison, WI, United States

**Keywords:** quercetin degradation, butyrate, *Eubacterium ramulus*, *Bacteroides thetaiotaomicron*, cross-feeding, starch

## Abstract

Consumption of flavonoids has been associated with protection against cardiovascular and neurodegenerative diseases. Most dietary flavonoids are subjected to bacterial transformations in the gut where they are converted into biologically active metabolites that are more bioavailable and have distinct effects relative to the parent compounds. While some of the pathways involved in the breakdown of flavonoids are emerging, little it is known about the impact of carbon source availability and community dynamics on flavonoid metabolism. This is relevant in the gut where there is a fierce competition for nutrients. In this study, we show that metabolism of one of the most commonly consumed flavonoids, quercetin, by the gut-associated bacterium *Eubacterium ramulus* is dependent on interspecies cross-feeding interactions when starch is the only energy source available. *E. ramulus* can degrade quercetin in the presence of glucose but is unable to use starch for growth or quercetin degradation. However, the starch-metabolizing bacterium *Bacteroides thetaiotaomicron*, which does not metabolize quercetin, stimulates degradation of quercetin and butyrate production by *E. ramulus* via cross-feeding of glucose and maltose molecules released from starch. These results suggest that dietary substrates and interactions between species modulate the degradation of flavonoids and production of butyrate, thus shaping their bioavailability and bioactivity, and likely impacting their health-promoting effects in humans.

## Introduction

Flavonoids are phenolic compounds produced by the secondary metabolism of plants. They are present in fruits, grains, and vegetables. Their basic structure consists of 15 carbon atoms arranged in three rings (A, B, and C). Their consumption is associated with a lower risk of suffering from cardiovascular and neurodegenerative diseases (Hertog et al., 1993; Letenneur et al., 2007; Rendeiro et al., 2015; Matias et al., 2016). Most polyphenols are poorly absorbed in the upper gastrointestinal tract (stomach, duodenum, jejunum, and ileum) and reach the colon where they are metabolized by the gut microbiota into more readily absorbable phenolic acids, increasing bioavailability of these biologically active compounds (Hollman et al., 1999; Manach et al., 2005).

Among the more than 8,000 different flavonoids characterized to date, quercetin is one of the most common in nature. It is found in apples, onions, red wine, tea, lettuce, and tomatoes and is extensively metabolized in the gastrointestinal tract (>90%; Hertog et al., 1992; Chen et al., 2005). Carbon dioxide is a major metabolite derived from quercetin metabolism in humans; a process that starts with the cleavage of the flavonoids C-ring by intestinal bacteria (Walle et al., 2001). Most members of the gut microbiota that are known to cleave the C-ring of quercetin belong to the Clostridia class; these include *Flavonifractor plautii, Eubacterium ramulus*, and *Eubacterium oxidoreducens* (Krumholz and Bryant, 1986; Braune et al., 2001; Schoefer et al., 2003). *E. ramulus* is a prevalent bacterial species commonly found in healthy subjects at levels ranging from 10^7^ to 10^9^ cells/ g of dry feces (Simmering et al., 1999). *E. ramulus* ferments glucose to butyrate, a major energy source of colonocytes that inhibits colon inflammation and carcinogenesis, and it has systemic effects lowering diet-induced insulin resistance (Roediger, 1982; Segain et al., 2000; Gao et al., 2009; Peng et al., 2009). *In vitro* studies indicate that *E. ramulus* requires glucose for the co-metabolization of quercetin (Schneider and Blaut, 2000). Degradation of this flavonoid results in the production of 3,4-dihydroxyphenylacetic acid (DOPAC), which has antiproliferative activity in colon cancer cells (Schneider and Blaut, 2000; Schneider et al., 2000; Gao et al., 2006). Nevertheless, glucose is rapidly absorbed in the small intestine and negligible amounts reach the colon, where *E. ramulus* resides (Low, 1988).

Dietary compounds that can impact microbial flavonoid metabolism in the colon are those that resist digestion in the upper gastrointestinal tract. Most carbohydrates that reach the lower gastrointestinal tract are of plant origin, including plant cell-wall and storage polysaccharides (Flint et al., 2008). Among these, the fraction of starch that escapes digestion in the small intestine; i.e., resistant starch, represents an important fermentation substrate that boosts bacterial flavonoid metabolism (Tousen et al., 2011) and butyrate production (Schwiertz et al., 2002). Starch that is incompletely digested in the upper digestive tract is naturally present in many foods, including bananas, rice, maize, and potatoes. For example, around 3% of hot potato starch and 12% of cold potato starch are resistant to digestion. It has been estimated that for individuals following a modern diet, the quantity of starch entering the colon is about 10% of starch intake, around 8–40 g/days (Cummings and Englyst, 1991). Previous work indicates that undigested starch enhances bacterial metabolism of daidzein, a soy isoflavone (Tousen et al., 2011). Furthermore, the abundance of the flavonoid-degrading bacterium, *E. ramulus*, is positively influenced by consumption of resistant starch (Simmering et al., 2002).

In order to get insights into the fate of flavonoids in the presence of polysaccharides in the multi-species environment of the gut, we evaluated in a simplified model of the gut microbiota the interactions between *E. ramulus*, which has a limited capacity to utilize polysaccharides, and *Bacteroides thetaiotaomicron*, which has the capacity to degrade many polysaccharides but it is unable to degrade the flavonoid, quercetin. We found that *B. thetaiotaomicron* liberates glucose and maltose from starch at levels that support the growth of *E. ramulus* and degradation of quercetin by this bacterium. Our results illustrate how cross-feeding between bacterial taxa can impact the metabolic fate of flavonoids in the gut.

## Materials and Methods

### Chemicals

Ammonium formate, 98% crystalline (Alfa Aesar, United States), EDTA ≥ 98.5% w/w (Sigma-Aldrich, United States), methanol HPLC-grade (Thermo Fisher Scientific, United States), ultrapure grade water purified to 18.1 MΩ⋅cm using a Barnstead water filtration system (Thermo Fisher Scientific, United States), potato starch (Sigma; 102954), and quercetin dihydrate, 97% w/w (Alfa Aesar, United States).

### Bacterial Strains and Culture Conditions

Frozen stocks of *E. ramulus* strain ATCC 29099 and *B. thetaiotaomicron* VPI-5482 were diluted 1:50 in 7N minimal medium, consisting of 50 mM MOPS⋅KOH (pH 7.2), 0.2% resazurin, 2 mM tricine, 0.025% tween 80, 20 mM C_2_H_3_NaO_2_, 20 mM NaCl, 14 mM NH_4_Cl, 0.25 mM K_2_SO_4_, 0.5 mM MgCl_2_⋅6H_2_O, 0.5 mM CaCl_2_⋅2H_2_O, 10 μM FeSO_4_⋅7H_2_O, 20 mM NaHCO_3_, 1 mM KH_2_PO_4_, 1 μg ml^-1^ vitamin K_3_, 1.9 μM hematin, 0.2 mM histidine, 8 mM L-cysteine, 1 × ATCC trace minerals, 1 × ATCC vitamin supplement, amended with 40 mM glucose. Media were filter-sterilized (0.22 μm pore diameter). Cultures were incubated with constant agitation at 37°C overnight (OD_600_ = 1.5–1.7 for *E. ramulus*, 1.3–1.5 for *B. thetaiotaomicron*), then washed thrice with 10 ml of anaerobic 7N medium without glucose inside an anaerobic chamber. All centrifugations steps were done in Hungate tubes at 3,000 rpm 5 min. After the final centrifugation, *E. ramulus* cell suspension was resuspended in a sixth of the initial volume and *B. thetaiotaomicron* in half of the initial volume to produce concentrated cell suspensions with equivalent number of cells for both (an overnight culture of *B. thetaiotaomicron* has about 3 times more cells than *E. ramulus*). For a typical assay, about 150 μl of cell suspension was added to a 10 ml medium (this corresponds to about 10^9^–10^10^ genome equivalents ml^-1^), cultures were grown at 37°C with agitation. Glucose was filter-sterilized and added at a final concentration of 40 mM. Starch was autoclaved and added at a final concentration of 1%. Quercetin dihydrate was used at a final concentration of 0.25 mg ml^-1^ in MilliQ water, autoclaved for 20 min, let cooled for 1 h with stirring and dispensed with stirring. Bacteria were handled inside an anaerobic chamber under an atmosphere of nitrogen (75%), carbon dioxide (20%), and hydrogen (5%).

### DNA Preparation

DNA extraction was performed as previously described with modifications (Turnbaugh et al., 2009). Briefly, 300 μl aliquot of cultures with starch as sole carbon source was mixed with a solution containing 500 μl of extraction buffer (200 mM Tris pH 8.0, 200 mM NaCl, 20 mM EDTA), 200 μl of 20% SDS, 500 μl of a mixture of phenol:chloroform:isoamyl alcohol (25:24:1, pH 7.9) and 1.2 mg of 0.1-mm diameter zirconia/silica beads (BioSpec Products, United States). The suspension was then subjected to 3 min of bead beating (BioSpec Products, United States) at room temperature (RT), spun at 8,000 rpm for 5 min at RT, and then 750 μl of the top layer was transferred to a 15 ml tube (BD Falcon 12 × 75 mm, #352063) for immediate column purification (NucleoSpin, Macherey-Nagel, Switzerland). Column binding buffer NTl was used at 2.5 vol., 3 washes with washing buffer NT3 were performed and final elution was done with 25 μl of low T_(10)_E_(0.1)_ buffer.

### Real-Time Quantitative PCR (qPCR)

Quantitative PCRs were performed using the SsoAdvanced universal SYBR Green supermix (2X) (BioRad, 172-5270-5275) and the BioRad CFX96 Real-Time PCR Detection System. Species-specific primers for the 16S rRNA gene were used at a final concentration of 0.4 mM. Primer sequences for *B. thetaiotaomicron* were 5^′^-GCAAACTGGAGATGGCGA-3^′^ and 5^′^-AAGGTTTGGTGAGCCGTTA-3^′^ (Tm 62.5°C) (Tong et al., 2011) and for *E. ramulus*, 5^′^-CGGTACCTGACTAAGAAGC-3^′^ and 5^′^-AGTTTCATTCTTGCGAACG-3^′^ (Tm: 55°C) (Rinttilä et al., 2004). Each culture was analyzed in triplicate. DNA extractions from pure cultures of *B. thetaiotaomicron* or *E. ramulus* were used to generate standard curves using 7 serial dilutions ranging from 442 ng ml^-1^ to 4.42 × 10^-4^ ng ml^-1^ and 235 mg ml^-1^ to 2.35 × 10^-4^ ng ml^-1^, respectively (quantified by the Qubit dsDNA HS assay). The qPCR run consisted of a denaturation step (95°C for 30 sec), an amplification step (35 cycles of 95°C for 10 s, Tm for 15 s and 60°C for 30 s), and a melting cycle (65–95°C 0.5°C increment 2–5 s/step).

### HPLC Analyses of Quercetin and Metabolites

Samples from 0 and 22 h cultures were thawed in ice, vortexed extensively and 400 μl were mixed with 1000 μl HPLC-grade methanol + 20 μM genistein as internal standard, the suspension was subjected to bead beating (BioSpec Products, Bartlesville) for 2 min at RT, then heated to 56°C for 20 min and spun for 10 min at 18, 000 g at RT. Then 1 ml of the supernatant was transferred to an HPLC vial and 200 μl of 10 mM ammonium formate/0.5 M EDTA buffer (pH 3.5) was added. Quercetin and its metabolites were analyzed using a Dionex UltiMate 3000 HPLC equipped with an LPG-3400 quaternary pump, a WPS-3000 analytical autosampler, a DAD-3000 diode array detector, and a FLD-3100 fluorescence detector. Separations were performed on a Kinetex 5 μm EVO C18, 100 Å, 250 × 4.6 mm column (Phenomenex, Torrance, CA, United States). Injection volumes were 5 μL. A flow rate of 1 ml min^-1^ was used throughout the 59 min run. The mobile phase was a binary gradient of (A) 10 mM ammonium formate and 0.3 mM ethylenediaminetetraacetic acid in water adjusted to pH 3.5 using concentrated HCl and (B) methanol. Solvents were vacuum filtered with 0.20 μm nylon membrane filters (Merk Millipore Ltd., Cork, Ireland). The gradient began at 5% B for 5 min, increased to 30% B over 30 min, increased to 95% B over 10 min, remained constant at 95% B for 5 min, decreased to 5% B over 2 min, and then re-equilibrated at 5% B for 7 min. Three-dimensional absorbance data were collected using the diode array detector and chromatograms of 280 nm absorbance were analyzed. Reportable values are shown in Supplementary Table S1 and an example chromatogram is shown in Supplementary Figure S1.

Samples were quantitated based on external calibration with injections of 10 μL over the linear range 1–125 μM for protocatechuic acid; 3,4-dihydroxyphenylacetic acid; 3,4-dihydroxyphenylpropionic acid; 3-hydroxybenzoic acid; 3-hydroxyphenylacetic acid; 3-(3-hydroxyphenyl) propionic acid; phenylacetic acid; quercetin; and genistein and 5–125 μM for benzoic acid. Some compounds could not be resolved by this method, namely 3-(3-hydroxyphenyl) propionic acid and phenylacetic acid.

### Gas Chromatography–Mass Spectrometry Measurements of Butyrate

Samples were processed as described before (Krautkramer et al., 2016). Briefly, an aliquot of 50 μl of cultures incubated for 12 h with starch and quercetin and *E. ramulus* monocultures with glucose and quercetin (control) were mixed with 20 mM of a butyric-d7 acid, 99.5 atom % D, CDN isotopes #D-171 as internal standard, acidified with 5 μl of 33% HCl, extracted twice with Diethyl Ether, then 60 μl of each sample was mixed with 2 μl of derivatizing reagent (*N*-Methyl-*N*-tert-butyldimethylsilyltrifluoroacetamide, MTBSTFA) and incubated at RT for 2 h. For detection, 1 μl of each sample was injected in a gas chromatography–mass spectrometry (GC-MS) instrument (Agilent 7890B/5977A GC/MSD), and an Agilent DB-1 ms column was used. Oven program was: initial temperature, 40°C for 2.25 min; then 20°C min^-1^ to 200°C; next 100°C min^-1^ to 300°C, maintained for 7 min.

### Determination of Glucose and Maltose

An aliquot of each culture with starch as sole carbon source was centrifuged at 11,000 ×*g* 5 min and glucose and maltose levels were determined by a colorimetric method (Maltose and Glucose Assay Kit; Sigma-Aldrich, MO, United States) at 0, 4, and 8 h of incubation following the manufacturer’s instructions. Standard curve: 2–10 nmol of glucose per well.

### Statistical Analysis

Data was analyzed using analysis of variance (ANOVA – Minitab 18.1). Differences considered significant at *p* < 0.05.

## Results

### *Eubacterium ramulus* Requires an Energy Source to Metabolize Quercetin

**FIGURE 1:**
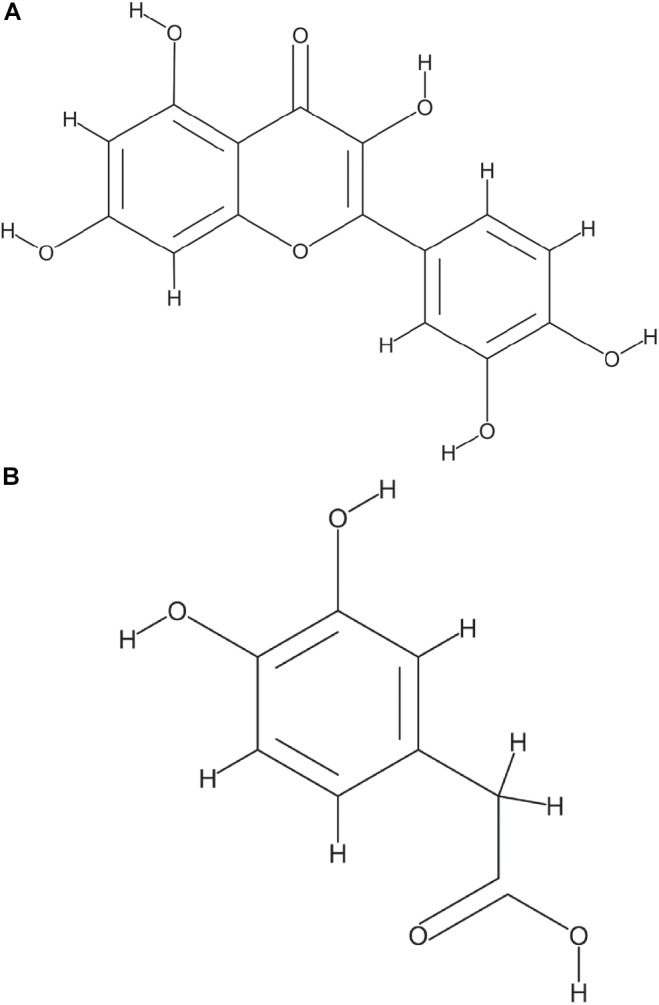
Structure of Quercetin **(A)** and 3,4-Dihydroxyphenylacetic acid (DOPAC), the main metabolite generated by *E. ramulus* from quercetin degradation **(B)** National Center for Biotechnology Information. PubChem Database, compound 5280343 and 547, respectively (https://pubchem.ncbi.nlm.nih.gov/).

**FIGURE 2:**
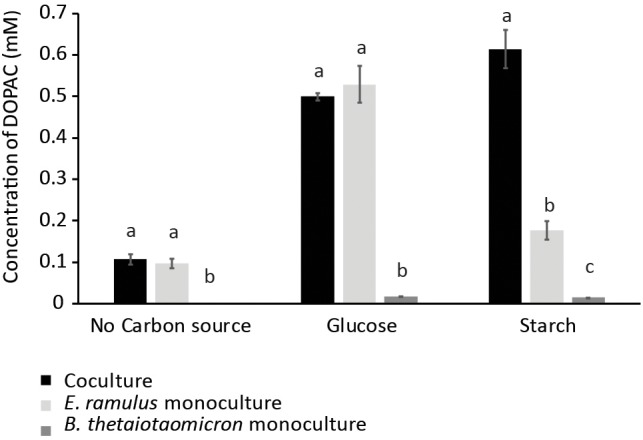
DOPAC concentration as measured by HPLC in cultures with no carbon source, glucose (0.7%), and starch (1%). Culture tubes were inoculated with only *B. thetaiotaomicron* or *E. ramulus* or both and incubated for 22 h. Error bars corresponds to 3 replicates. Different letters above bars indicate significant difference between type of culture at *p* < 0.05 according to Least Significant Difference (LSD).

**FIGURE 3:**
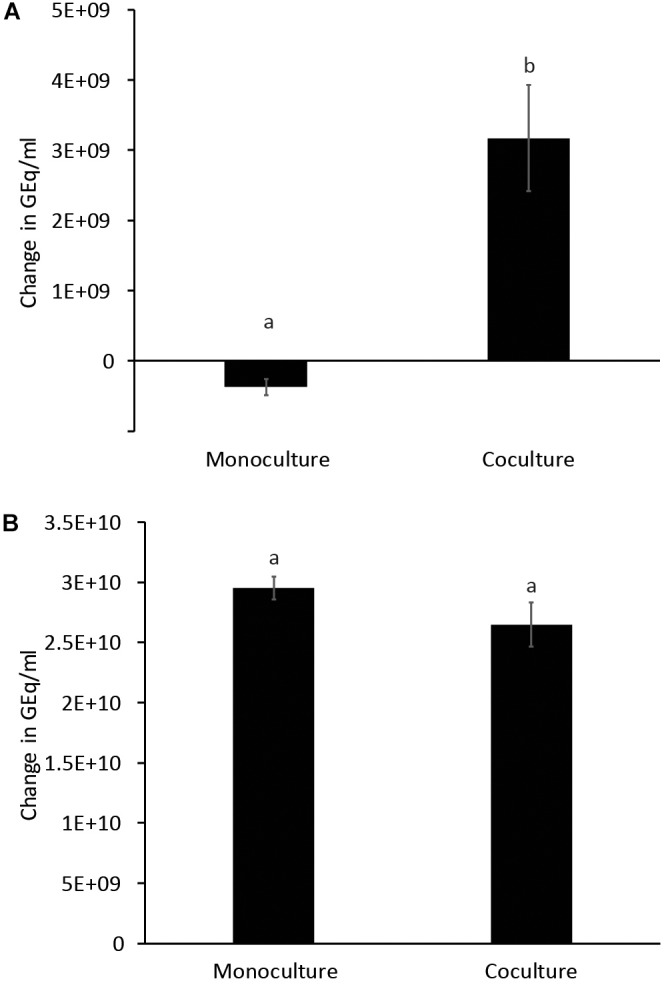
Growth of *E. ramulus* **(A)** and *B. thetaiotaomicron* **(B)** in cultures with 1% starch as carbon source. GEq, Genome equivalents. Change in Geq/ml between time 0 and 8 h. Results from independent experiments are presented in Supplementary Table S2. Different letters above bars indicate significant differences between type of culture at *p* < 0.05 according to LSD, data from 2 independent experiments (with 2–3 replicates each).

Glucose and starch were evaluated for their capacity to promote quercetin degradation by *E. ramulus*. The structure of quercetin is shown in Figure 1A. We used media with no addition of a carbon source as a negative control. We found that glucose stimulates the degradation of quercetin by *E. ramulus* and the production of 3,4-dihydroxyphenylacetic acid (DOPAC) (Figure 1B) as the main metabolite derived from quercetin (Figure 2 and Supplementary Table S1), as previously described (Schneider and Blaut, 2000). In the presence of glucose *E. ramulus* also generates high levels of butyrate (Table 1). We assessed different concentrations of glucose in order to determine the lowest concentration of this monosaccharide required for the co-metabolization of 0.8 mM of quercetin. Quercetin degradation was checked by visual inspection; i.e., disappearance of the quercetin from the test tube, which is insoluble and has a yellow color. We also quantified quercetin and the main degradation product, DOPAC, by HPLC at the end of the experiment (22 h). We found that concentrations of glucose above 0.3 mM are required to stimulate detectable quercetin degradation (Supplementary Figure S2). Additionally, we observed a decrease in the population of *E. ramulus* when no carbon source was added but quercetin (Supplementary Table S2), suggesting cell death. Accordingly, under these conditions little production of DOPAC was detected (Figure 2). Monocultures of *E. ramulus* supplemented only with starch did not show growth (Figure 3A and Supplementary Table S2), as expected they accumulated little butyrate (Table 1) and DOPAC after 22 h of incubation (Figure 2). There were no signs of quercetin degradation after 4 days of incubation (data not shown). *E. ramulus* did not grow on starch with or without quercetin (Supplementary Figure S3).

**Table 1:** Butyrate concentrations in monocultures and co-cultures of *E. ramulus* and *B. thetaiotaomicron*.

Culture and carbon source	Butyrate (mM)
*E. ramulus* monoculture with glucose	7.09 ± 0.40
*E. ramulus* monoculture with starch	0.22 ± 0.07^a^
*B. thetaiotaomicron* monoculture with starch	0.01 ± 0.0005^b^
Coculture with starch	3.19 ± 0.06^c^


*Average values for 3 replicates. Different letters after standard deviation indicate significant difference between type of culture (only starch cultures) at *p* < 0.05 according to LSD.*

### *Bacteroides thetaiotaomicron* Starch Utilization Enhances Butyrate Production and Quercetin Degradation by *Eubacterium ramulus*

We examined whether the presence of the versatile polysaccharide-metabolizing bacterium, *B. thetaiotaomicron*, influenced the degradation of quercetin and production of butyrate by *E. ramulus*. We incubated both species with quercetin and starch individually and in co-culture. We did not observe significant degradation of quercetin by either species in monoculture (Figure 2). However, a marked appearance of DOPAC (Figure 2) and enhanced growth of *E. ramulus* was observed in co-cultures (Figure 3A and Supplementary Table S2). Growth of *E. ramulus* was not enhanced in co-cultures when glucose was the sole carbon source (Supplementary Figure S4). Additionally, while butyrate was not produced by either species in monoculture incubated with starch, co-cultures accumulated high levels of butyrate, ∼44% of the amount produced by *E. ramulus* monocultures with 40 mM glucose (Table 1). On the other hand, the presence of *E. ramulus* did not change significantly the growth yield of *B. thetaiotaomicron* incubated with 1% starch (Figure 3B and Supplementary Table S2).

### *Bacteroides thetaiotaomicron* Releases Glucose and Maltose From Starch

Lastly, we hypothesized that *B. thetaiotaomicron* promotes *E. ramulus* metabolism by releasing free glucose from starch. We quantified free glucose in cultures with starch as the sole carbon source (Table 2). We found that *B. thetaiotaomicron* releases free glucose at levels that are higher than what we found is necessary to stimulate quercetin degradation (Supplementary Figure S2), whereas co-cultures accumulated approximately 50% of the glucose of *B. thetaiotaomicron* monocultures (*p* < 0.05) (Table 2). Additionally, we detected maltose in monocultures of *B. thetaiotaomicron* incubated with starch at a concentration of 0.2 mM. ( ± 0.04 mM) at 4 h and 0.8 mM ( ± 0.1 mM) at 8 h of incubation. In co-cultures, there were lower concentrations of maltose, 0.08 mM ( ± 0.02 mM) after 4 h of incubation and 0.4 mM ( ± 0.2 mM) after 8 h. Accordingly, maltose also stimulated the degradation of the flavonoid by *E. ramulus* in monoculture (data not shown). Altogether these results suggest that *E. ramulus* uses glucose and maltose released by *B. thetaiotaomicron* from starch to grow, produce butyrate and to degrade the flavonoid.

**Table 2:** Concentration of glucose liberated from starch by *E. ramulus* or *B. thetaiotaomicron* (monocultures) or both organisms in coculture.

Culture	Free glucose (mM)		
	0 h	4 h	8 h
*E. ramulus* monoculture	0.02 ± 0.004^a^	0.02 ± 0.01^a^	0.06 ± 0.05^a^
*B. thetaiotaomicron* monoculture	0.03 ± 0.01^a^	1.29 ± 0.43^b^	8.29 ± 2.76^b^
Coculture	0.02 ± 0.01^a^	0.68 ± 0.33^c^	4.62 ± 1.70^c^


*Average values for 2 independent experiments with 2 replicates each. Different letters after parenthesis indicate significant difference between treatments at each time point at *p* < 0.05 according to LSD.*

## Discussion

Here we evaluated how the utilization of a common carbohydrate in human diet, starch, by a member of the Bacteriodetes phylum changes the dynamics of flavonoid degradation and production of butyrate by *E. ramulus*. We found that metabolization of starch by *B. thetaiotaomicron* enhanced these processes in the Firmicute via cross-feeding of glucose and maltose released from the carbohydrate. Mahowald et al. (2009) observed in gnotobiotic mice co-colonized with *Eubacterium rectale* and *B. thetaiotaomicron* that *E. rectale* is better able to access nutrients and upregulates genes in the central carbon and nitrogen pathways in the presence of the *B. thetaiotaomicron* (Mahowald et al., 2009). One explanation for this is the observation that *B. thetaiotaomicron* releases simple saccharides when digesting complex carbohydrates that then *Eubacterium* can access. We have observed that *B. thetaiotaomicron* can enhance the growth of *E. ramulus* when growing on different oligo and polysaccharides (e.g., inulin, FOS, and arabinogalactan; data not shown), however, the most striking stimulation was on starch. *B. thetaiotaomicron* possess membrane-associated amylase activity, encoded by *susG*, which may allow the release of products of starch breakdown to the extracellular medium (Shipman et al., 1999), however, not all bacteria can access these public goods (Rakoff-Nahoum et al., 2014). Cross-feeding of starch-derived metabolites has also been reported for *Bifidobacterium adolescentis*, this bacterium generates resources that can be used by butyrate producers including *Roseburia* sp., *Eubacterium hallii*, and *Anaerostipes caccae.* This cross-feeding involves end-products (lactate or acetate) of *B. adolescentis* starch fermentation and potentially products released by partial hydrolysis of starch likely to be malto-oligosaccharides (Belenguer et al., 2006), however, it is not clear whether glucose is released from starch by *B. adolescentis*. In our studies, supplementation of acetate to the media (20 mM), in the absence of a usable carbohydrate, did not prompt the degradation of quercetin or the production of butyrate by *E. ramulus*.

In this work, we evaluated a soluble form of starch. Soluble starch could reach the colon entrapped in non-soluble cell-wall particles which can be released when cellulose-degrading microorganisms (e.g., *Ruminococcus* spp. or *Enterococcus* spp.) act on them releasing the soluble part. It is also possible that cellulose-degrading microorganisms could release free glucose, however, the capacity to degrade cellulose in humans seems to be limited (Robert and Bernalier-Donadille, 2003; Flint et al., 2008). *B. thetaiotaomicron* has a great ability to ferment soluble starch while its ability to ferment resistant starch depends on the type of resistant starch and treatment. *In vitro* experiments show an efficiency of >90% for autoclaved or boiled high amylopectin corn starch but <1% for raw high amylose corn starch. Thus, in the gut other players that ferment resistant starch more efficiently may potentially play an important role in providing resources for cross-feeding. It is worth noting that several butyrate-producers including *E. ramulus* do not have the capacity to degrade starch; non-etheless resistant starch is recognized as a butyrogenic substrate (Schwiertz et al., 2002), thus we suggest that the acquisition of luminal products of starch breakdown (e.g., glucose and maltose) by butyrate-producing species may be important for the production of butyrate.

Several studies indicate that quercetin has antibacterial activity (Rauha et al., 2000; Vaquero et al., 2007). Quercetin increases the permeability of certain bacteria to ions (Mirzoeva et al., 1997). Some species of bacteria have defense mechanisms against quercetin. For example, the plant root-colonizer, *Pseudomonas putida*, has an efflux pump, TtgABC, that has a high affinity for quercetin, as well as for certain antibiotics (Terán et al., 2006). Cellular targets also include enzymes like DNA gyrase and D-alanine:D-alanine ligase, essential for DNA replication and the assembly of peptidoglycan precursors, respectively, in these enzymes quercetin recognizes the conserved ATP-binding region and compete with ATP (Plaper et al., 2003; Wu et al., 2008). Some sugar transporters depend on ATP (Kaplan and Hutkins, 2003) thus the ability of using starch when quercetin was not present was evaluated for *E. ramulus*. Under these conditions *E. ramulus* was also unable to use starch. Thus, our data indicates that *E. ramulus* is unable to perform the initial breakdown of starch but possess the ability of using public goods generated by the breakdown of this substrate (Rakoff-Nahoum et al., 2014). In the intestine, the ability to degrade quercetin may create a temporal niche where *E. ramulus* can access nutrients (e.g., luminal glucose) that are in the vicinity of flavonoids and that other flavonoid-sensitive bacteria cannot access while the concentration of the flavonoid is still high.

Microbial-derived biologically active metabolites have a major impact on host’s health. Quercetin is one of the most abundant flavonoids, however, it is still not clear whether its colonic degradation is beneficial for the host since the parent compound and bacterial products derived from its degradation all have bioactivity. Studies that explore the extent to which degradation of quercetin is beneficial for the host are needed. For this goal, carbohydrates that promote more or less the degradation of the flavonoid can be useful. For example, it has been shown that fructooligosaccharides administered to the diet accelerate the use of the flavonoids rutin, quercitrin and quercetin in cecal contents of rats relative to animals supplemented with non-fermentable fiber (Juśkiewicz et al., 2011). The characterization of gut microbes able to metabolize flavonoids will make possible in the future to stratify subjects in clinical trials based on the flavonoid-degrading capacity of their gut microbiotas. Furthermore, understanding how interpersonal or disease-associated differences in gut microbial metabolism of flavonoids impact the potential benefits associated with their consumption, and identifying biomarkers for these processes will help nutritionists formulate dietary recommendations that are matched by the metabolic potential of a subject’s gut microbiota with the ultimate goal of optimizing food function efficacy.

## Author Contributions

GR-C and FR conceived and planned the experiments. GR-C, MD, and XL carried out the experiments. GR-C, AA-C, BB, and FR contributed to the interpretation of the results. AA-C and FR helped to supervise the project. GR-C wrote the manuscript with input from all authors. All authors discussed the results and contributed to the final manuscript.

## Conflict of Interest Statement

The authors declare that the research was conducted in the absence of any commercial or financial relationships that could be construed as a potential conflict of interest.
