# Supplementary material for: Bacteroides thetaiotaomicron Starch Utilization Promotes Quercetin Degradation and Butyrate Production by Eubacterium ramulus
Source: Front Microbiol. 2019 May 29;10:1145. doi: 10.3389/fmicb.2019.01145 (PMC6548854; doi:10.3389/fmicb.2019.01145)
Supplement: Supplementary file 1 [file Data_Sheet_1.docx]

Supplementary Material


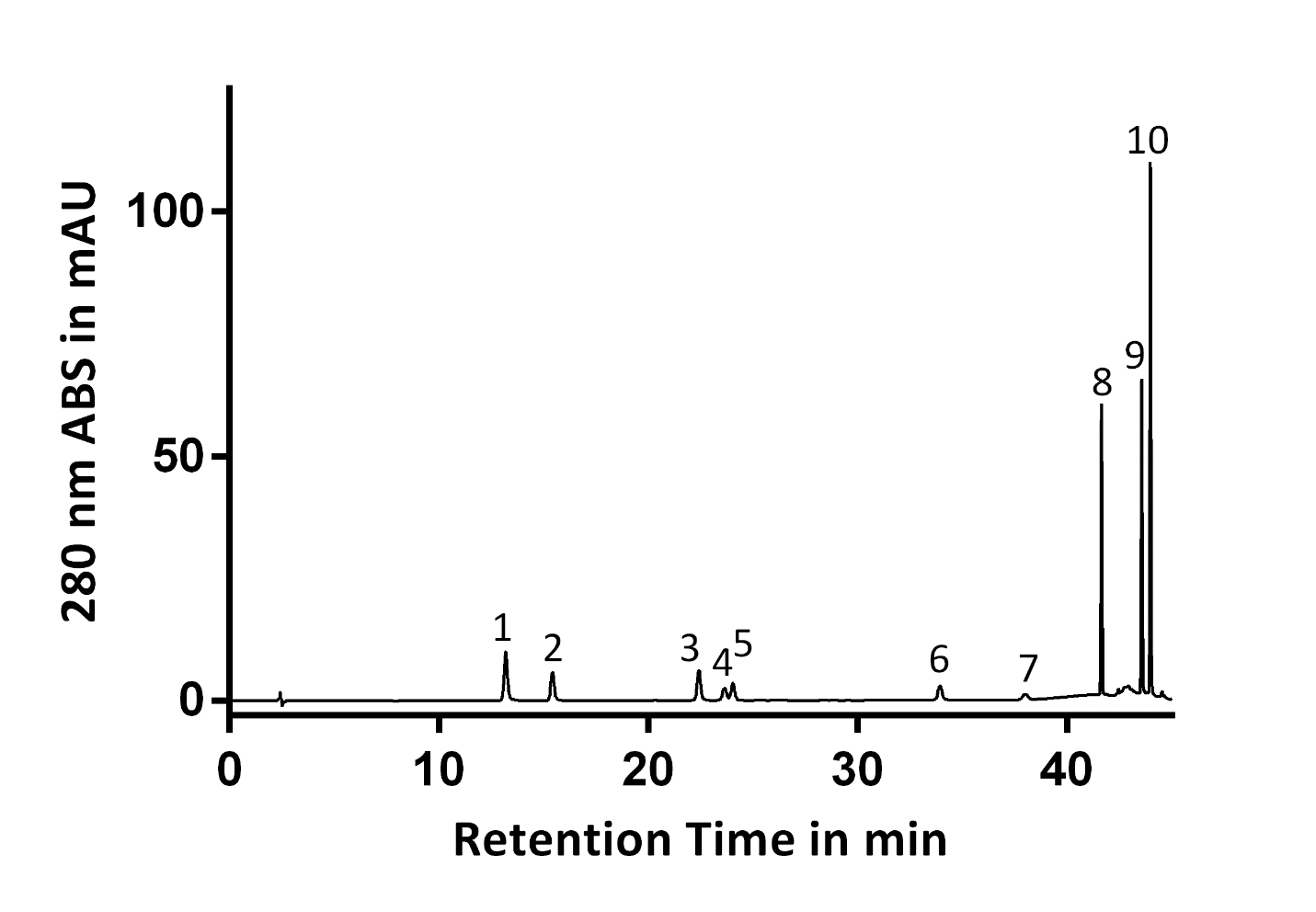


**Figure S1**. Chromatogram for calibration of standards. Reference compounds had the following retention times: 13.2 min for 3,4-dihydroxybenzoic acid (PCA, 1), 15.4 min for 3,4-dihydroxyphenylacetic acid (DOPAC, 2), 22.4 min for 3,4-dihydroxyphenylpropionic acid (3), 23.7 min for 3-hydroxybenzoic acid (4), 24.1 min for 3-hydroxyphenylacetic acid (5), 33.9 min for 3-(3-hydroxyphenyl)propionic acid + phenylacetic acid (6), 38.0 min for benzoic acid (7), 41.7 min for rutin (8), 43.6 min for quercetin (9), and 44.0 for genistein (10). Concentration of each analyte 100 µM.


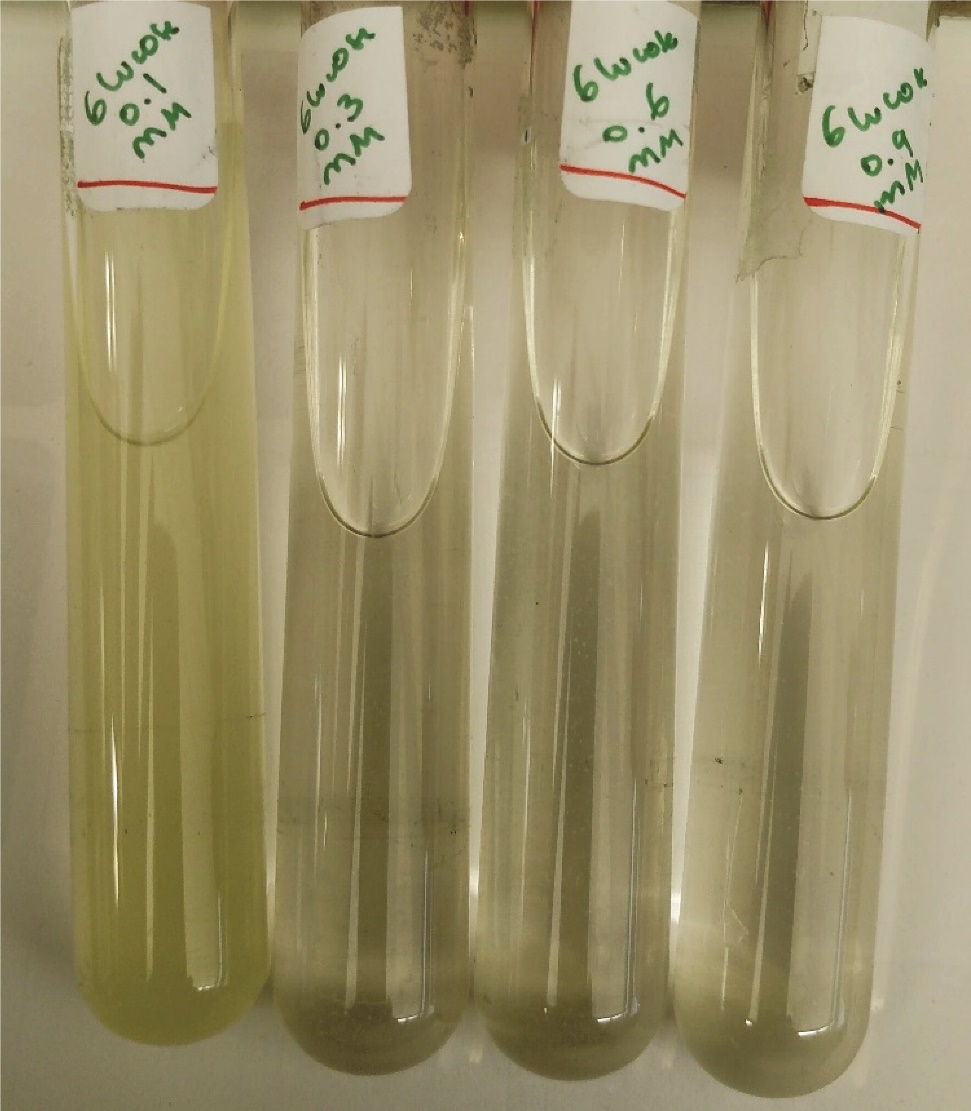


**Figure S2.** Quercetin degradation assay inoculated with *E. ramulus* with different concentrations of glucose (0.1, 0.3, 0.6, and 0.9 mM) at 22 h of incubation. Degradation was monitored through the visualization of the yellow color of quercetin. No transformation of quercetin, yellow; transformation, transparent. Tubes correspond to representative results from 3 replicates.


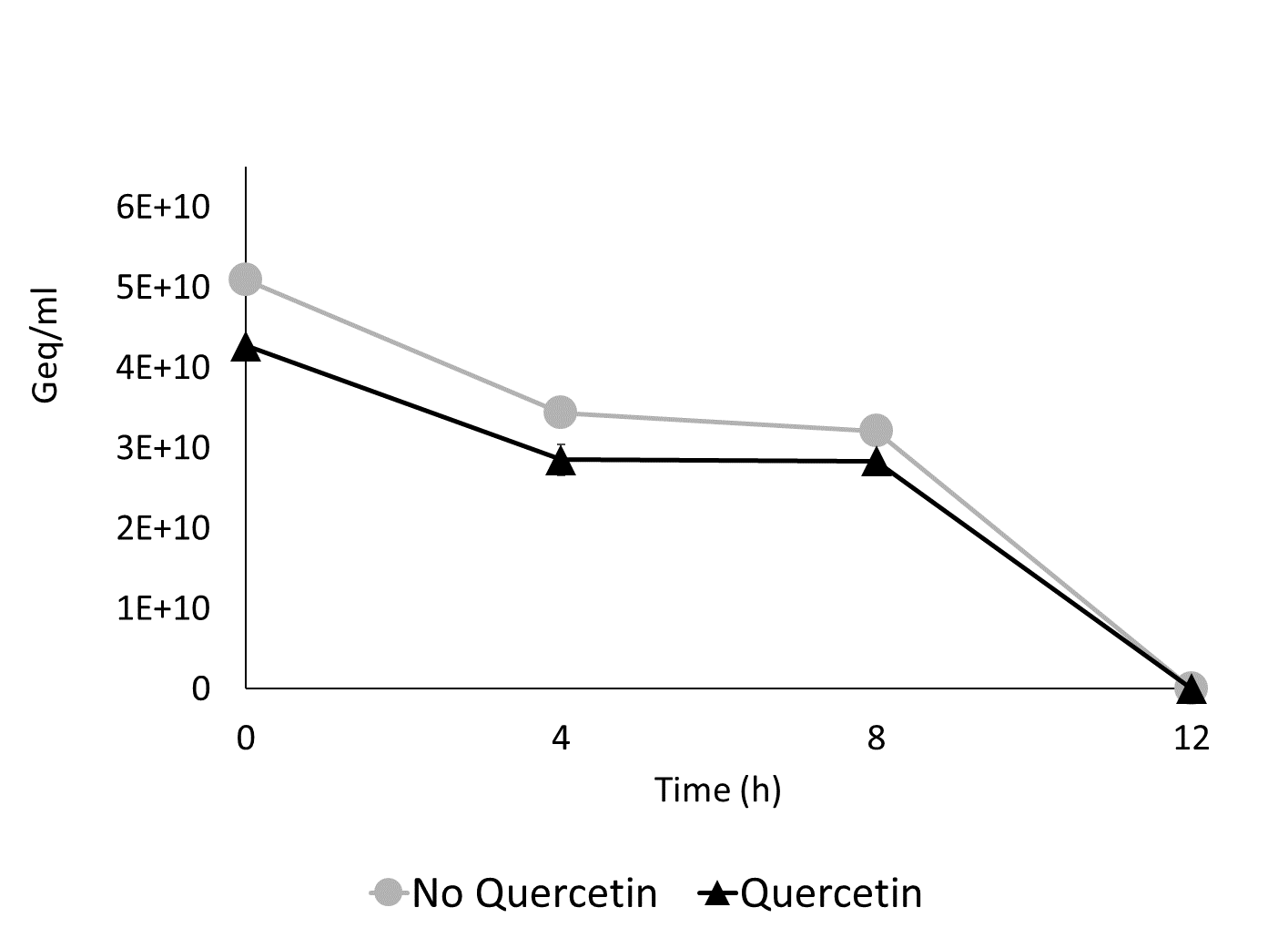


**Figure S3.** Lack of growth of *E. ramulus* in media supplemented with 1 % starch as carbon source with (black) and without Quercetin (gray). GEq, Genome equivalents.

**
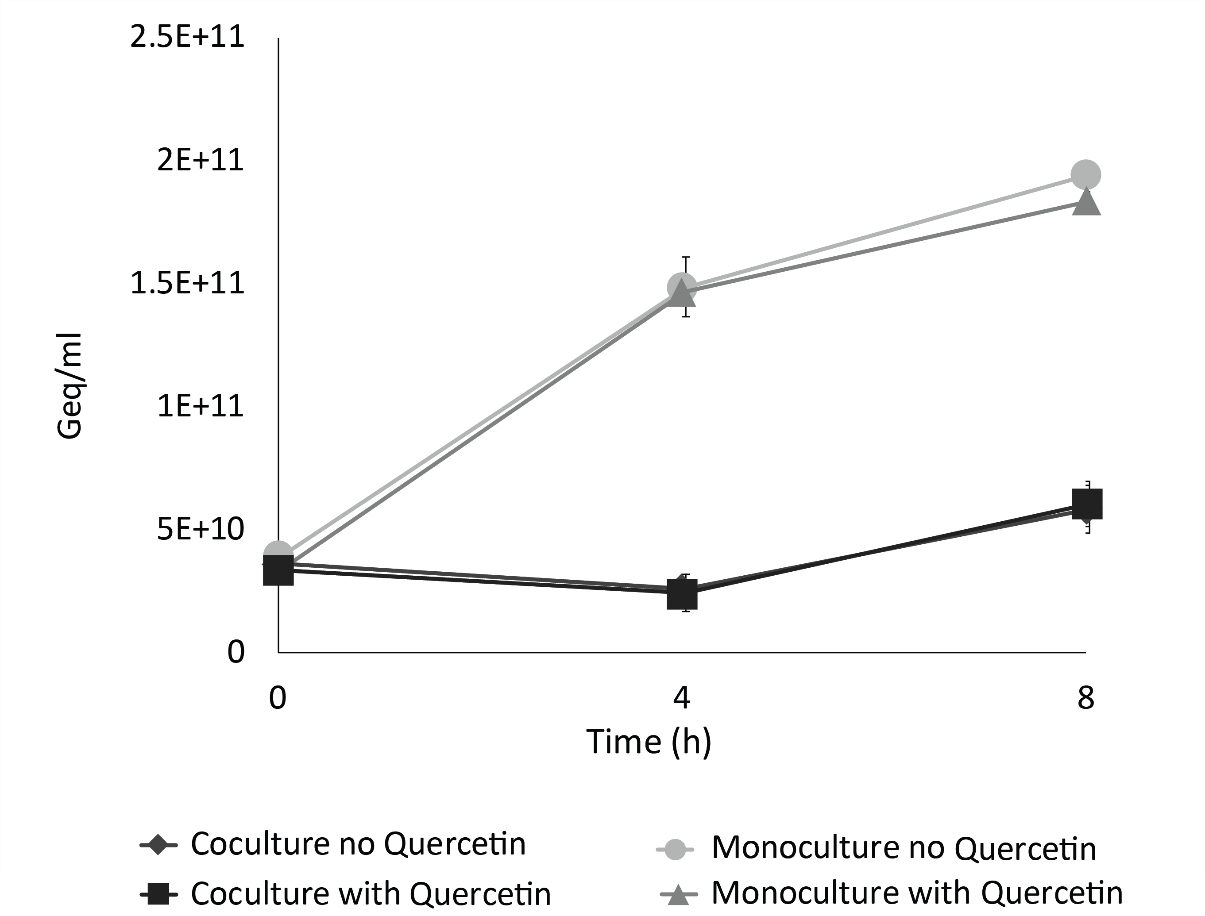
**

**Figure S4.** Growth of *E. ramulus* in monocultures and cocultures supplemented with 40 mM of glucose as carbon source with and without quercetin. GEq, Genome equivalents.

**Table S1**. HPLC reportable values for Quercetin and related compounds in monocultures and cocultures at 22 h.

| **Substrate** | **Culture** | **Q^q^** | **D^d^** | **C^c^** | **E^c^** | **F^f^** | **H^h^** | **I + J^i^** | **K^k^** |
| --- | --- | --- | --- | --- | --- | --- | --- | --- | --- |
| No Carbon source | *B. thetaiotaomicron* | 155.47 | **<0.99** | <4.99 | <0.99 | <0.99 | <0.99 | <4.99 | <4.99 |
| No Carbon source | *B. thetaiotaomicron* | 190.82 | **<0.99** | <4.99 | <0.99 | <0.99 | <0.99 | <4.99 | <4.99 |
| No Carbon source | *B. thetaiotaomicron* | 197.20 | **<0.99** | <4.99 | <0.99 | <0.99 | <0.99 | <4.99 | <4.99 |
| Glucose | *B. thetaiotaomicron* | 201.67 | **3.93** | 5.11 | <0.99 | <0.99 | <0.99 | <4.99 | <4.99 |
| Glucose | *B. thetaiotaomicron* | 199.52 | **4.13** | 5.50 | <0.99 | <0.99 | <0.99 | <4.99 | <4.99 |
| Glucose | *B. thetaiotaomicron* | 179.65 | **4.10** | <4.99 | <0.99 | <0.99 | <0.99 | <4.99 | <4.99 |
| Starch | *B. thetaiotaomicron* | 226.80 | **3.63** | <4.99 | <0.99 | <0.99 | <0.99 | <4.99 | <4.99 |
| Starch | *B. thetaiotaomicron* | 183.53 | **3.60** | 5.08 | <0.99 | <0.99 | <0.99 | <4.99 | <4.99 |
| Starch | *B. thetaiotaomicron* | 192.92 | **3.63** | 5.03 | <0.99 | <0.99 | <0.99 | <4.99 | <4.99 |
| No Carbon source | *E.ramulus* | 170.27 | **26.25** | <4.99 | <0.99 | <0.99 | <0.99 | <4.99 | <4.99 |
| No Carbon source | *E.ramulus* | 172.08 | **22.29** | <4.99 | <0.99 | <0.99 | <0.99 | <4.99 | <4.99 |
| No Carbon source | *E.ramulus* | 168.88 | **20.73** | <4.99 | <0.99 | <0.99 | <0.99 | <4.99 | <4.99 |
| Glucose | *E.ramulus* | 18.44 | **113.76** | 10.55 | <0.99 | <0.99 | <0.99 | <4.99 | <4.99 |
| Glucose | *E.ramulus* | 2.98 | **132.62** | 9.81 | <0.99 | <0.99 | <0.99 | <4.99 | <4.99 |
| Glucose | *E.ramulus* | 6.06 | **131.26** | 10.34 | <0.99 | <0.99 | <0.99 | <4.99 | <4.99 |
| Starch | *E.ramulus* | 162.91 | **47.63** | 6.36 | <0.99 | <0.99 | 1.21 | <4.99 | <4.99 |
| Starch | *E.ramulus* | 203.09 | **41.97** | 5.67 | <0.99 | <0.99 | <0.99 | <4.99 | <4.99 |
| Starch | *E.ramulus* | 173.60 | **36.92** | <4.99 | <0.99 | <0.99 | <0.99 | <4.99 | <4.99 |
| No Carbon source | Coculture | 171.92 | **21.89** | <4.99 | <0.99 | <0.99 | <0.99 | <4.99 | <4.99 |
| No Carbon source | Coculture | 165.69 | **27.51** | <4.99 | <0.99 | <0.99 | <0.99 | <4.99 | <4.99 |
| No Carbon source | Coculture | 166.26 | **26.97** | <4.99 | <0.99 | <0.99 | <0.99 | <4.99 | <4.99 |
| Glucose | Coculture | 25.56 | **117.58** | 12.21 | <0.99 | <0.99 | <0.99 | <4.99 | <4.99 |
| Glucose | Coculture | 22.79 | **117.77** | 12.56 | <0.99 | <0.99 | <0.99 | <4.99 | <4.99 |
| Glucose | Coculture | 18.70 | **121.08** | 11.93 | <0.99 | <0.99 | <0.99 | <4.99 | <4.99 |
| Starch | Coculture | 34.33 | **138.66** | 6.42 | <0.99 | <0.99 | <0.99 | <4.99 | <4.99 |
| Starch | Coculture | 39.18 | **141.16** | 6.05 | <0.99 | <0.99 | <0.99 | <4.99 | <4.99 |
| Starch | Coculture | 25.34 | **158.65** | 6.37 | <0.99 | <0.99 | <0.99 | <4.99 | <4.99 |

^q^Q, Quercetin; ^d^D, 3,4-dihydroxyphenylacetic acid (DOPAC, in bold); ^c^C, 3,4-dihydroxybenzoic acid (PCA); ^e^E, 3,4-dihydroxyphenylpropionic acid; ^f^F, 3-hydroxybenzoic acid; ^h^H, 3-hydroxyphenylacetic acid; ^i^I+J, 3-(3-hydroxyphenyl) propionic acid + phenylacetic acid; ^k^K, benzoic acid. Concentration in µM. A number after the symbol “<” indicates less than the minimum detectable value.

**Table S2**. Change in Genome equivalents of *E. ramulus* and *B. thetaiotaomicron* in cultures with 1 % starch as carbon source (experiment independent from the one shown in Fig. 2) and no carbon source.

| Experiment |  | Change in Geq/ml | |
| --- | --- | --- | --- |
|  |  | No Carbon Source | Starch |
| Monoculture | *E. ramulus* | -8.63×10^8^ ±4.7×10^8^ *^a^* | -3.17E×10^9^ ±5.3×10^9^ *^b^* |
|  | *B. thetaiotaomicron* | - | 2.07×10^11^ ±1.8×10^10^ *^a^* |
| Coculture | *E. ramulus* | *-* | 2.20E×10^10^ ±2.6×10^9^ *^b^* |
|  | *B. thetaiotaomicron* | *-* | 2.78×10^11^ ±1.5×10^10^ *^a^* |

GEq, Genome equivalents

*^a^* Change in Geq/ml between time 0 h and 8 h.

*^b^* Change in Geq/ml between time 0 h and 12 h.
